# A commentary on ‘Survival benefit and impact of adjuvant chemotherapy following systemic neoadjuvant chemotherapy in patients with resected pancreas ductal adenocarcinoma: a retrospective cohort study’

**DOI:** 10.1097/JS9.0000000000000960

**Published:** 2023-12-04

**Authors:** Furui Zhong, Chuanbo Xieb, Xuefeng Peng, Jinlong Luo, Hua Yang

**Affiliations:** a Department of General Surgery of Huidong, Zigong Fourth People’s Hospital; b Department of Ultrasound, Zigong Hospital of Woman and Children Healthcare, Zigong, Sichuan Province, People’s Republic of China

The combination of upfront pancreatectomy followed by adjuvant chemotherapy (AC) remains the established treatment for resectable pancreatic ductal adenocarcinoma (PDAC), as confirmed by several randomized trials^1–3^. In recent years, neoadjuvant chemotherapy (NAC) has gained attention as a viable treatment strategy for borderline resectable and locally advanced PDAC. This is due to its significant advantages in tumor downstaging, enhancing R0 resection rates, and ultimately improving overall survival (OS)^4,5^. However, limited and inconclusive evidence exists regarding the potential benefits of AC in patients undergoing pancreatic cancer resection following NAC. van Roessel *et al*.^6^ conducted a multicenter retrospective cohort study to address this issue, demonstrating that only patients with pathology-proven node-positive disease had improved survival when they received AC after NAC and pancreatic cancer resection. Subsequently, Sugawara *et al*.^7^ demonstrated a significant survival benefit associated with AC following NAC and resection in PDAC patients, consistent across various subgroups related to age, pathological T category, and resection margin.

In a recent article in the *International Journal of Surgery*, Pu *et al*.^8^ performed a retrospective, propensity-matched analysis using Surveillance, Epidemiology and End Results (SEER) data to evaluate the efficacy of AC in patients with resected pancreatic cancer after NAC. They included 1589 patients and concluded that systemic AC was associated with longer median OS (30.0 vs. 25.0 months, *P*=0.002) and cancer-specific survival (33.0 vs. 27.0 months, *P*=0.004) compared to the non-AC group. Subgroup analysis revealed that patients ≤65 years and those with node-positive disease (pathological N1 category) had a lower mortality when treated with AC. This large cohort study provides robust evidence supporting the clinical benefit of systemic AC in patients with resected PDAC after NAC.

Despite the propensity score matching, there are common issues associated with retrospective analysis of SEER data. *First*, as discussed, crucial data, such as surgical margin status and pathological response to multiagent NAC were unavailable. Indeed, patients who did not achieve an R0 resection and complete pathological response are more likely to receive AC post-surgery. All of these confounding factors have a significant correlation with patient prognosis, potentially impacting the study’s conclusions. *Second*, this study found that the OS benefit of AC was specifically observed in patients aged ≤65 years. This observation may be attributed to their superior performance status and chemotherapy tolerance. Performance status is a crucial predictor of postoperative mortality in various surgical procedures, yet this study lacks a corresponding indicator^9^. Moreover, data on postoperative complications are inadequate. Patients who experience postoperative complications are more likely to delay or even forgo AC, resulting in a worse prognosis. *Finally*, the time span of the included SEER data (2006–2019), would have certainly led to significant variations in treatment regiments. However, this study lacks detailed regimens and durations of NAC and AC, which severely limits the clinical generalizability of these findings.

Overall, we commend the authors for their remarkable efforts in assessing the survival benefit of systemic AC in patients undergoing NAC and resection. Considering the inherent limitations of SEER data, further prospective and large-scale studies targeting specific protocols and populations are necessary to confirm these findings.

## Ethical approval

Not applicable for this study.

## Sources of funding

This work was supported by key science and technology Project of Zigong (Nos.2022ZCYGY12).

## Author contribution

F.Z.: study design and writing; C.X. and X.P.: critical review; H.Y.: study supervision.

## Conflicts of interest disclosure

The authors declare that they have no conflicts of interest.

## Research registration unique identifying number (UIN)

1. Name of the registry: not applicable.
2. Unique identifying number or registration ID: not applicable.
3. Hyperlink to your specific registration (must be publicly accessible and will be checked): not applicable.

## Guarantor

Furui Zhong and Hua Yang.
